# Learning to use electronic health records: can we stay patient-centered? A pre-post intervention study with family medicine residents

**DOI:** 10.1186/s12875-017-0640-2

**Published:** 2017-05-26

**Authors:** Cédric Lanier, Melissa Dominicé Dao, Patricia Hudelson, Bernard Cerutti, Noëlle Junod Perron

**Affiliations:** 1 Department of Community Medicine, Primary Care and Emergency Medicine, Geneva University Hospitals, rue Gabrielle Perret-Gentil, CH-1211 Geneva, Switzerland; 2 Primary care unit, University of Geneva, Centre Médical Universitaire de Genève, Geneva, Switzerland; 3 Faculty of Medicine, University of Geneva, Geneva, Switzerland

**Keywords:** Electronic health record, Patient-Physician relation, Computer, Communication skills, Training

## Abstract

**Background:**

The Electronic Health Record (EHR) is now widely used in clinical encounters. Because its use can negatively impact the physician-patient relationship, several recommendations on the “patient-centered” use of the EHR have been published. However, the impact of training to improve EHR use during clinical encounters is not well known. The aim of this study was to assess the impact of training on residents’ EHR-related communication skills and explore whether they varied according to the content of the consultation.

**Methods:**

We conducted a pre-post intervention study at the Primary Care Division of the Geneva University Hospitals, Switzerland. Residents were invited to attend a 3-month training course that included 2 large group sessions and 2–4 individualized coaching sessions based on videotaped encounters. Outcomes were: 1) residents’ perceptions regarding the use of EHR, measured through a self-administered questionnaire and 2) objective use of the EHR during the first 10 min of patient encounters. Changes in practice were measured pre and post intervention using the Roter interaction analysis system (RIAS) and EHR specific items.

**Results:**

Seventeen out of 27 residents took part in the study. Participants used EHR in about 30% of consultations. After training, they were less likely to consider EHR to be a barrier to the physician-patient relationship, and felt more comfortable using the EHR. After training, participants increased the use of signposting when using the EHR (pre: 0.77, SD 1.69; post: 1.80, SD3.35; p 0.035) and decreased EHR use when psychosocial issues appeared (pre: 24.5% and post: 9.76%, p < 0.001).

**Conclusions:**

This study suggests that training can improve residents’ EHR-related communication skills, especially in situations where patients bring up sensitive psychosocial issues. Future research should focus on patients’ perceptions of the relevance and usefulness of such skills.

## Background

The Electronic Health Record (EHR) is now widely used in clinical encounters and its use is promoted by national incentive programs [1–7]. The EHR improves the quality of biomedical data gathering and reduces the number of medical errors [8–13]. It also facilitates the sharing of medical information with the patient [14–16].

The literature shows that patients and physicians are mainly satisfied with the use of the EHR [13, 17–21]. However, some patients worry about the loss of confidentiality [18, 22] while some physicians express concerns about the negative impact of the EHR use on the physician-patient interaction [23–25].

Behavioral changes linked to the use of the EHR include the following: increased time spent with the EHR during the encounter, especially during the first minutes of the encounter [26], increased moments of silence and a decrease in visual interaction between the physician and the patient [13, 27–31]. Such behaviours tend to distract physicians from picking up verbal or non verbal cues expressed by their patients [32]. Indeed, the time spent looking at the computer screen appears to be inversely correlated with physicians’ interest for patient’s psychosocial and emotional discourse [28–30].

Based on such observations, experts in medical communication issued several recommendations on the use of the EHR during the encounter in order to stay patient-centered (Table 1) [28, 33, 34]. Recommendations focus on physician’s verbal and nonverbal communication skills especially during the first few minutes of the encounter. Indeed, physician-patient communication at the beginning of the encounter is particularly important as it sets the stage for a good relationship with the patient, and contributes to identifying the patient’s emotional state and concerns, and to establishing a partnership with the patient [35, 36]; the way the EHR is used clearly affects the opening of the encounter [26]. Experts also highlight the importance of shifting away from the computer when patients express sensitive psychosocial issues [33, 34]. Other non communication elements also include physicians’ typing and computer skills, spatial arrangement of the computer and screen, and personal style of EHR use [37–40].

**Table 1:** Recommendations on how to use the electronic health record (EHR). Adapted from [33, 34]

• To open the EHR before the patient enters the consultation room	
•To set the agenda of the consultation before using the EHR/the keyboard • To explore the patient’s agenda • To negotiate the agenda by taking into account the patient’s agenda	
• To allow the patient to have a visual access to the screen/EHR during the clinical encounter (when possible)	
• To face the patient most of the time	
• To signpost the use of EHR (to summarize what the patient said, to announce what is done with the EHR: documentation, EHR reading, etc.…)	
• To use verbal and non verbal attitudes to show the patient that the physician’s attention is directed to the EHR or the patient (visual and/or verbal link)	
• To involve the patient in reading the information or results displayed on the screen (to give information to help understanding)	
• To stop using the EHR when patient expresses emotions or psychosocial issues (to stop typing, to look at the patient, listen, express verbal empathy)	
• To use appropriate time sets to type (when the patient put on/off his cloths before or after the physical examination)	

Experts recommend integration of EHR skills in the undergraduate medical curriculum [41–44]. However, only a few studies have assessed the impact of training on the use of such recommendations and these report contrasting results. In a control-group study with first year medical students and standardized patients, Morrow and al. showed that a training course involving role-play increased the use of EHR-related communication skills such as introducing oneself before turning towards the computer, introducing the computer in the triadic relationship, alerting the patient verbally when the doctor turns his/her attention to the computer, and sharing visual information on the screen in the intervention group [45]. Reis et al. compared the impact of two different training formats (traditional lecture vs simulation-based) on resident-patient-EHR communication in a primary care training setting. Performances and attitudes improved in both groups. However, the simulation-based group evaluated the training experience more highly than did the traditional lecture group [46]. Han et al. reported a controlled pre-post intervention study in which medical students demonstrated more patient-centered skills than the control group after having attended an online self-study module on how to preserve the patient-centered relationship while using the EHR [47]. Silverman et al. showed that a specific course on ergonomic computer-use helped medical students to use the EHR in a more patient-centered way during clinical encounters with standardized patients [48].

However, these interventional studies rarely involved real patients, assessed only short term effects (2–3 weeks) [27] and did not specifically study the impact of training on EHR use according to the content of the encounter.

The aim of our study was to assess the impact of training on EHR-related communication skills of residents with real patients during the first 10 min of the clinical encounter. We chose to focus on the first 10 min because we have observed that our residents tend to use the EHR mainly at the beginning of the encounter. In particular, we wanted to explore how EHR use changed when patients introduced psychosocial issues.

## Method

### Design, setting and participants

A pre-post study was conducted at the Primary Care Division of the Geneva University Hospitals, Switzerland between April and September 2013. The Primary Care Division provides approximately 13’000 medical consultations a year (a majority of follow-up consultations for chronically ill patients) to a diverse urban population, and is also a training center for 40 residents who spend 12 to 24 months training in primary care at the end of their general internal medicine residency training (after 2–4 years of hospital training) before moving to independent practice.

#### EHR implementation

In January 2012, a new electronic health record (EHR) was developed for primary care consultations, and all physicians in the division are now required to document patient encounters using this EHR. This new EHR is located within the hospital electronic health record, and reflects a problem-oriented medical structure [49]. Data can be entered in either free text fields or via pre-structured fields developed based on the French version of the International Classification of Primary Care (ICPC) [50].

#### Residents’ and patients’ perceptions regarding EHR use

In October 2012, as part of a needs assessment, a group discussion with 12 residents was conducted in order to explore their perceptions and difficulties regarding the use of the EHR during clinical encounters. They perceived several advantages such as rapid access to and visibility of the information documented in EHR for all health providers, facilitated billing and EHR-based drug prescription. They also reported several difficulties such as the need to master typing skills, the possible loss of confidentiality, and a negative impact on physician-patient communication which they described as altered and more distant, with a loss of visual contact. However, a small phone survey conducted by CL among a random sample of 20 patients who regularly attended the clinic for chronic conditions showed that they were not disturbed by the use of EHR (100% of the patients reported that their physician’s use of EHR was beneficial and none was disturbed by such use).

Based on these results, we decided to develop and assess a training program on how to maintain patient-centered communication when using the EHR during clinical encounters. Residents from two clinics of the division, mainly new residents, were invited to take part in the study: the general primary care clinic (*n* = 21) and the primary care clinic for asylum seekers (*n* = 6).

### Intervention

**Fig. 1:**
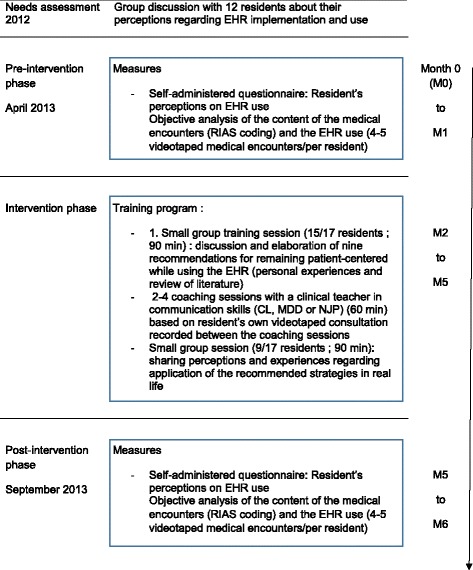
Overview of the intervention conducted and evaluation measures collected among residents

The 3-month training course included two large group sessions and 2–4 individual supervisions based on residents’ own videotaped encounters (Fig. 1). During the first large group session which lasted 90 min, residents were asked to suggest and share strategies considered to be useful for overcoming the difficulties of EHR use during consultations. Based on this discussion and a review of the literature [33, 34], we elaborated nine recommendations for remaining patient-centered while using the EHR during the entire length of clinical encounters, which were subsequently provided to residents (Table 1).

During the next three months, pairs of residents had 2 to 4 1-h coaching sessions under the supervision of a clinical teacher in communication skills (CL, MDD or NJP). Residents were asked to videotape 1–2 new clinical encounters between the coaching sessions (those videos were not analysed for the study). At the beginning of the session, residents were first asked to reflect on their strengths and weaknesses on the basis of the nine recommendations developed during the large group session and provided during the coaching session; they then watched segments of videotaped clinical encounter (a segment chosen by participants or from the beginning, depending on their preferences) and analysed their EHR related behaviors together (the 2 residents and the teacher) ; individual difficulties were addressed through role-play followed by feedback. The focus depended on residents’ needs but often included signposting when using the EHR (telling the patient what you are doing when you shift your attention to the computer) and stopping EHR use when patients expressed emotions or psychosocial issues. Objectives for improvement were identified and documented from one session to the next on a paper portfolio displaying the nine recommendations. The number of sessions varied according to the degree of improvement shown by participants but did not exceed 4.

During the second large group session which took place after three months, the residents involved in the training were asked to share their perceptions and experiences regarding the recommended strategies.

### Data collection and outcome measures

Outcomes measures were

1. Residents’ perceptions regarding the use of the EHR before and after the training intervention

Three weeks before and three weeks after the 3-month training period, participants were asked to fill in an 11-item, self-administered questionnaire (5-point Likert scales) about their perceptions regarding the use of the EHR during their clinical encounters (Table 3). This questionnaire was developed based on a review of the literature [23–25, 51–54].

2) Video-based analysis of the clinical encounters

Participants were also asked to videotape 3–4 of their own encounters during a half day three weeks before and three weeks after the training period. Eligible encounters were those conducted in French or English and without the presence of a third person (e.g. family member, interpreter). Before beginning the consultation, participants asked eligible patients to provide a written informed consent to being videotaped during the entire length of the consultation. The analysis was then performed only for the first ten minutes.

#### Content of the first 10 min of the clinical encounter before and after the training intervention

The content of the physician-patient verbal interaction was coded using the Roter interaction analysis system (RIAS), which is a well-known and validated tool used to capture patterns of verbal communication [55]. The physician and patient interaction was first coded using 7 content categories: 1) medical 2) therapeutic 3) lifestyle 4) psychosocial 5) positive talk 6) emotional talk 7) partnership. Other content codes, such as negative and social talk, were not used due to their minimal presence during clinical encounters. Within each content category and for each speaker, the type of utterance was also coded (closed or open-ended question, information-giving, counseling). Coding was performed by an experienced coder from a Canadian group who developed the French version of the RIAS, using MEDICODE software [56].

#### Objective use of the EHR during the first 10 min of the clinical encounter before and after the training intervention

The physicians’ use of the EHR was coded using a scale we developed specifically for this study, based on initial observations and analysis of 15 videotaped encounters by the investigators, and a review of the literature [26, 28, 30, 40, 57, 58]. Coded behaviors included: use of signposting (telling the patient what you are doing when you shift your attention to the computer) when using the EHR and use of the keyboard and/or the screen with or without verbal or visual contact with the patient. Contact was coded as present when the physician displayed verbal, non verbal or visual contact with the patient at intervals of less than 5 s. Contact was coded as absent if the physician showed none of these behaviors during periods of 5 s or more (Table 2). This length of time was based on a study showing that pauses of at 5 s or more were more likely to break the conversation and to cause a change in the topic of the conversation [13, 59]. In order to evaluate whether EHR use varied according to the segment of the consultation (opening, history taking, physical examination, explanation/counseling, closing), a sub-sample of videotaped encounters (12 in the pre-intervention and 11 post-intervention) was coded during the entire length of the consultation.

**Table 2:** Coding scheme for the electronic health (EHR) record communication

EHR use	Description
EHR signposting^a^	Signposting that he/she is starting to type or look at the screen
No EHR use	No use of keyboard or screen
EHR use with patient link	Verbal, non verbal (nodding) or visual link with the patient at least 1x during 5 s while • Typing on the keyboard • Looking at the screen • Typing on the keyboard and looking at the screen
EHR use without patient link	No verbal, non verbal or visual link with the patient during > 5 s while • Typing on the keyboard • Looking at the screen • Typing on the keyboard and looking at the screen

^a^Telling the patient what you are doing when you shift your attention to the computer

The EHR coding, linked to both the RIAS utterances and to time (in seconds) was performed by DR on an excel file.

#### Patterns of EHR use

We coded patterns of EHR use as: “low” users (<30% of utterances/time linked to EHR use during the first ten minutes), “medium” users (30–40%) and “high” users (>40% of utterances/time linked to EHR use during the first ten minutes) on the basis of previous research [40].

Intra-rater reliability of RIAS coding, calculated on the basis of 18% videotaped encounters showed 95.5% of stability in utterance cutting and 80.5% of convergence in utterance coding and intraclass correlation coefficient was 0.97. The coder was regularly informed about the convergence rates. The categories for which the rate of disagreement was more than 20% were then discussed with another experienced coder in order to achieve agreement. Since all encounters were coded by the same rater, inter-rater agreement was not an issue in this study. However, a previous study has documented good inter-rater reliability of the RIAS [60].

Inter-rater reliability of EHR coding, calculated on the basis of 10% of the videotaped clinical encounters and performed by CL and NJP, was good (intraclass correlation coefficient = 0.91).

The study was approved by the Geneva University Hospital’s research ethics committee.

### Statistical Analysis

Descriptive statistics (frequency tables and relative percentages, means, standard deviations) were used to describe the residents’ self-perceptions regarding the training course and its impact on their EHR use, content of physician-patient interaction and the use of EHR during videotaped encounters.

The residents’ self-perceptions about the impact of the training were compared using paired t tests. The frequencies and relative frequencies of the use of EHR were compared using Chi-squared tests. All tests were performed with a significance level of 0.05. All analyses were run on R 2.15.3 (the R Foundation for Statistical Computing), and TIBCO Spotfire S + ® 8.1 for Windows (TIBCO Software Inc).

## Results

Seventeen residents (63%) accepted to participate after having been contacted by email: 14 worked in the general primary care clinic and 3 in the primary care clinic for asylum seekers; 10 (59%) were female and mean age was 35 years old (SD 5.2). Five residents (29%) had obtained postgraduate certification in general internal medicine and fourteen (82%) had attended medical school in Switzerland. We did not record reasons for non-participation, but these included reluctance to be videotaped, lack of time/availability or interest.

Residents attended 2.8 (SD 0.57) individualized supervisions; fifteen residents (88%) took part in the first small group session and 9 (53%) in the second session. Reasons for non-attendance included being on vacation, on call or on sick leave.

1. Residents’ perceptions regarding the use of the EHR

Residents generally felt the training helped improve their EHR-related skills and after the training course they were less likely to consider the EHR as a barrier to the physician-patient relationship (Table 3). However, they did not think the training had much impact on more general aspects of the patient-provider relationship (e.g. time dedicated to the patient, interest in their complaints, interruptions of patient talk…).

**Table 3:** Residents’ self-perceptions about the impact of the training

Residents’ self-perceptions	Number of residents *n* = 17		
(Likert 1 = completely disagree; −5 = completely agree)	Pre Mean (SD)	Post Mean (SD)	p
Training evaluation			
The training met my needs	-	4.11 (0.60)	
The training improved my communication skills related to the use of EHR	-	4.53 (0.52)	
The training improved my « general » communication skills	-	3.88 (0.78)	
I would recommend this training	-	4.43 (0.65)	
Self-perceptions on EHR use			
I feel comfortable using the EHR during the encounter	3.00 (1.23)	3.76 (1.20)	0.04
I have good typing skills	2.65 (1.12)	3.18 (0.88)	0.07
Using the EHR during the encounter increases the time dedicated to the patient	2.29 (1.26)	2.71 (1.16)	0.26
Using the EHR impacts negatively on the physician-patient relationship	3.65 (1.32)	3.41 (0.94)	0.51
Using the EHR decreases the visual link with the patient	4.00 (1.21)	3.81 (1.05)	0.57
Using the EHR is a barrier for the physician-patient relationship	3.53 (1.01)	3.00 (1.00)	0.03
I make the patient feel comfortable	4.18 (0.73)	4.41 (0.50)	0.22
I show interest for the patient’s ideas	4.29 (0.77)	4.41 (0.51)	0.54
I pay attention to the patient	4.06 (0.75)	4.29 (0.69)	0.26
I let the patient talk without interruption	3.59 (0.94)	3.76 (0.97)	0.55

During the debriefing session, residents described what they learned about maintaining patient-centeredness when using the EHR, including: involving patients when using the EHR, indicating to the patient when using the EHR, documenting key words instead of writing sentences, maintaining contact with the patient during typing phases, and use strategic times to type.

2) Video-based analysis of the clinical encounters

One hundred fourty-two videotaped encounters were analysed (pre-intervention: *n* = 73 and post-intervention: *n* = 69).

### Content of the first 10 min of the clinical encounter

The content of the first 10 min of the clinical encounter focused mainly on utterrances in the following categories: medical (pre : 17% ; post : 18%), therapeutic (pre : 11% ; post : 13%), positive talk (pre : 22% ; post : 21%) or partnership (pre : 18% ; post : 20%). The lifestyle (pre: 3.5% ; post : 2.7%), psychosocial (pre : 6.6% ; post : 4.2%) and emotional talk (pre : 2.9% ; post : 2.8%) categories occurred less often during the first 10 min of the clinical encounter.

### Objective use of the EHR during the first 10 min of the clinical encounter

In general, residents used the EHR about one-third of the utterances/time during the first ten minutes of the clinical encounter both intervention phases (pre: 27.9/26.2%; post: 30.4/28.2%), and used it more during medical and therapeutic talk and less for lifestyle or psychosocial matters (Table 4). EHR use with visual/verbal contact was 4 times more frequent than without visual/verbal contact.

**Table 4:** Use of Electronic Health Record (EHR) during the first ten minutes of consultation pre and post-training

Overall EHR use	All consultations (*n* = 142)		
	Pre (*n* = 73)	Post (*n* = 69)	
	Mean (SD)	Mean (SD)	p
EHR Signposting	0.77 (1.69)	1.80 (3.35)	0.04
	All utterances (*n* = 29011)		
	Pre (*n* = 15181) n (%)	Post (*n* = 13830) n (%)	p
EHR total use^a^	4237 (27.9)	4207 (30.4)	<0.01
• With link^a^	3215 (21.2)	3180 (23)	<0.01
• Without link^a^	800 (5.3)	726 (5.3)	0.96
Medical condition and therapeutic regimen^b^	1232 (28.5)	1322 (30.6)	0.04
Medical condition (without counseling)^b^	774 (29.8)	790 (31.8)	0.12
Therapeutic regimen (without counseling)^b^	458 (26.6)	532 (28.9)	0.14
Lifestyle and psychosocial^b^	307 (24.8)	114 (15.3)	<0.01
Lifestyle (without counseling)^b^	99 (25.5)	62 (24.8)	0.91
Psychosocial (without counseling)^b^	208(24.5)	52 (10.5)	<0.01
Positive talk^b^	821 (25.1)	871 (29.7)	<0.01
Emotional talk^b^	92 (21.2)	97 (25.5)	0.18
Partnership^b^	697 (25)	745 (27)	0.10

^a^% calculated with numbers of utterances while using EHR/all utterances

^b^% calculated with number of utterances per RIAS category while using EHR/number of utterances per RIAS category

After training, participants increased the use of signposting when using the EHR (Table 4). Although participants increased their use of the EHR after training (Table 4), it occurred only for medical or therapeutic talk (without verbal/visual contact) and positive talk (with verbal/visual contact). The use of the EHR significantly decreased during discussion of psychosocial issues (Table 4). Such changes were observed among different types of users of EHR (low: pre 57 utterances (10.6%) vs post 20 (4.6%) *p* = 0.01; medium: pre 27 (30.0%) vs post 12 (26.7%) *p* = 0.01; high: pre 162 (43.2%) vs post 24 (27.0%) *p* = 0.01).

Analysis of entire consultations confirmed that EHR was used more often during the opening and history taking phases (55 and 66% of the pre- and post-intervention phases) than during the explanation and counseling phases (40 and 32% of the pre- and post-intervention phases).

### Patterns of EHR use

Five residents (29.4%) were high EHR users, 5 (29.4%) were medium users and 7 (41, 2%) were low users.

## Discussion

Our study shows that before the training intervention, residents used the EHR about 30% of the time/utterances during the initial few minutes of the encounter and more often for medical than for psychosocial topics. They also used the EHR four times more often while maintaining visual/verbal contact.

Significant changes after the intervention included: more signposting of EHR use, increased link with patients while using EHR, more use of EHR related to medical and therapeutic topics and less use associated with psychosocial issues. Residents reported feeling more comfortable using EHR in the consultation, and perceived the EHR as less of a barrier to communication.

Our results confirm those previously published in adult primary care outpatient contexts. Two recent systematic reviews about the impact of the EHR use on the patient-doctor interaction report a mean use of EHR of 32% of the visit time with a range from 12 to 55% with experienced physicians using it less often [13, 27]. Most of the studies (76%) were conducted in adult primary care outpatient clinics but similar results were observed in specialities and in different clinical settings. Very few studies analysed the use of EHR among residents [13, 27]. One study which analysed screen gaze and use of typing among US residents showed increased EHR use (including both screen gaze and typing) among more experienced residents: 43 and 18% respectively among 3rd year residents vs 30 and 9% among 1st year residents. Screen gaze shared by both residents and patients was stable (10%) [61]. To our knowledge, no study analysed EHR use with or without maintaining visual or verbal contact with the patient.

After training, our study participants felt more comfortable using the EHR and were less likely to feel that it interfered with the doctor-patient interaction. These findings are in line with studies conducted in encounters with standardized patients, and support the claim that specific training can improve EHR acceptance among medical students, residents and physicians [46, 48]. This is of importance since physicians are more reluctant than patients to use EHRs and are usually less satisfied than patients when using computers during clinical encounters [13, 27, 62]. The fact that increased EHR use among residents correlates with patient’s dissatisfaction supports the need to train residents on the EHR use in a patient-centered way [61].

The training also impacted on residents’ behaviour by increasing the use of signposting. Transition statements such as “signposting” are known to help the patient understand the structure and the meaning of the consultation, increase his/her participation and implication in the encounter and inform him/her when a new topic is introduced [35, 63, 64]. It is particularly important when using the EHR since the frequency of transitions statements is inversely correlated with the screen gaze [30]. Our results show that signposting, a strategy largely recommended by experts while using the EHR, can be taught effectively [13, 28, 33, 34].

We also found that the training course led to decreased use of the EHR when discussing psychosocial issues with patients, regardless of the resident’s typing style (low, medium or high EHR users). This result is of interest since most experts recommend avoiding computer use when a psychosocial topic is addressed [33, 34].

Why residents increased their use of EHR during both medical and “positive talk” during the first ten minutes of the consultation post training remains unclear. Positive talk includes utterances such as laughter, agreement approvals and compliments and is considered to contribute to build the relationship [55, 65, 66]. Residents may still have difficulties postponing EHR use when biomedical issues are discussed. Residents may also use positive talk to enhance relationship-building while using the EHR. Further research should evaluate whether these changes also occur during the entire length of the encounter.

### Limitations

Our study has several limitations. We were not able to conduct a controlled or randomized study given the small number of residents who accepted to take part into the study and the pre-post intervention design may threaten the validity of the results given a possible learning effect on use of EHR over time. However, since behavioral changes essentially focused on elements emphasized during training, such changes may be more related to the training than to natural progression over time. The timing and spatial disposition of the videotaping did not allow us to analyse the impact of training for two of the recommendations regarding EHR use: EHR opening was not systematically recorded and the screen position was not always entirely visible on all videotaped encounters. We also made no distinction between visual and verbal contact. Although previous studies have adopted different approaches [30, 67, 68], it is still unclear if verbal contact has the same “value” as visual contact during the physician-patient interaction. The importance of verbal and non verbal communication which has been emphasized by several authors should be further explored when related to EHR use [69, 70]. We did not assess patients’ perceptions regarding the use of EHR, because prior studies have shown that patients were already very satisfied with their physician communication styles and that the introduction of EHRs did not change their satisfaction [17, 21, 62, 71]; given the results of our small phone survey on the use of EHR among chronically ill patients from our clinic, we did not expect to find any difference in patient’s perceptions. We only analysed EHR use during the first ten minutes of the clinical encounter, and we may have missed important EHR related behaviors later in the consultations as well as changing patterns of EHR use. We based our study on the use of one type of EHR program and different EHR programs may yield different EHR behaviors. Finally, the fact that participants themselves asked their patients to participate and knew they were being videotaped may represent a limitation (through selection bias and Hawthorne effect).

## Conclusion

Paying attention to both the patient and the computer is challenging and can modify the patient-physician relationship. Given the increasing use of EHRs in health care settings, physicians must find the best way to perform EHR-related tasks without negatively affecting the physician-patient relationship. This study suggests that training can improve residents’ EHR-related communication skills, especially in situations where patients bring up sensitive psychosocial issues. EHR-related communication skills training should be integrated at all stages of medical training given the ongoing development of EHR in the health care system. More research is needed to further explore the EHR-related behaviours that patients expect or value from their doctor, in order to give more scientific evidence to expert recommendations on how and when to use the EHR. It would also be of interest to analyse which factors related to patients, physicians and computers influence the use of EHR during the clinical encounter.

## Abbreviations

EHR: Electronic Health Record
ICPC: International Classification of Primary Care
RIAS: Roter interaction analysis system
